# Chemical Composition, Biological Activities and In Silico Analysis of Essential Oils of Three Endemic *Prangos* Species from Turkey

**DOI:** 10.3390/molecules27051676

**Published:** 2022-03-03

**Authors:** Gokhan Zengin, Mohamad Fawzi Mahomoodally, Evren Yıldıztugay, Sharmeen Jugreet, Shafi Ullah Khan, Stefano Dall’Acqua, Adriano Mollica, Abdelhakim Bouyahya, Domenico Montesano

**Affiliations:** 1 Department of Biology, Science Faculty, Selcuk University, Konya 42130, Turkey; gokhanzengin@selcuk.edu.tr; 2 Department of Health Sciences, Faculty of Medicine and Health Sciences, University of Mauritius, Réduit 80837, Mauritius; f.mahomoodally@uom.ac.mu (M.F.M.); sharmeenjugs@gmail.com (S.J.); 3 Deparment of Biotechnology, Science Faculty, Selcuk University, Konya 42130, Turkey; eytugay@gmail.com; 4 Department of Pharmacy, Abasyn University, Peshawar 25000, Pakistan; shafiullahpharmd@gmail.com; 5 Department of Pharmaceutical and Pharmacological Sciences, University of Padova, Via Marzolo 5, 35131 Padova, Italy; stefano.dallacqua@unipd.it; 6 Department of Pharmacy, University “G. d’Annunzio” of Chieti-Pescara, 66100 Chieti, Italy; a.mollica@unich.it; 7 Laboratory of Human Pathologies Biology, Department of Biology, Faculty of Sciences, Mohammed V University in Rabat, Rabat 10106, Morocco; boyahyaa-90@hotmail.fr; 8 Department of Pharmacy, University of Naples Federico II, Via D. Montesano 49, 80131 Naples, Italy

**Keywords:** *Prangos*, essential oil, chemical composition, antioxidant, enzyme inhibition, molecular docking

## Abstract

In this study, the essential oils (EOs) obtained from three endemic *Prangos* species from Turkey (*P. heyniae*, *P. meliocarpoides* var. *meliocarpoides*, and *P. uechtritzii*) were studied for their chemical composition and biological activities. *β*-Bisabolenal (12.2%) and caryophyllene oxide (7.9%) were the principal components of *P. heyniae* EO, while *P. meliocarpoides* EO contained sabinene (16.7%) and *p*-cymene (13.2%), and *P. uechtritzii* EO contained *p*-cymene (24.6%) and caryophyllene oxide (19.6%), as the most abundant components. With regard to their antioxidant activity, all the EOs were found to possess free radical scavenging potential demonstrated in both DPPH and ABTS assays (0.43–1.74 mg TE/g and 24.18–92.99 mg TE/g, respectively). Additionally, while no inhibitory activity was displayed by *P. meliocarpoides* and *P. uechtritzii* EOs against both cholinesterases (acetyl- and butyryl-cholinesterases). Moreover, all the EOs were found to act as inhibitors of tyrosinase (46.34–69.56 mg KAE/g). Molecular docking revealed elemol and *α*-bisabolol to have the most effective binding affinity with tyrosinase and amylase. Altogether, this study unveiled some interesting biological activities of these EOs, especially as natural antioxidants and tyrosinase inhibitors and hence offers stimulating prospects of them in the development of anti-hyperpigmentation topical formulations.

## 1. Introduction

Essential oils (EOs) are recognized for their exceptional medicinal value and are considered among the most attractive and potent plant-derived products. Eos, also referred to as ethereal oils, are volatile and odorous oils present in only 10% of the plant kingdom and are stored in plants in special brittle secretory structures, for instance glands, secretory hairs, secretory ducts, secretory cavities or resin ducts. EOs have been used as perfumes, flavors in foods and beverage ingredients, or to heal both the body and mind since ages and even today they continue to be of paramount importance [1].

In fact, many studies have focused on the pharmacological and cosmeceutical potentials of EOs such as antioxidant, antimicrobial, anticancer, anti-inflammatory, antiaging and antimelanogenic amongst many others [2,3,4]. Accordingly, growing evidences of the health benefits of these natural essences have prompted researchers to further investigate EOs and their individual components. Additionally, their mechanisms of action have been elucidated with respect to their biological activities [5,6,7,8], which so far are showing promising prospects.

The genus *Prangos* (Apiaceae) consists of 45 worldwide species and the genus has a wide distribution area ranging from Portugal to Tibet [9]. The genus is represented by 19 taxa, including 19 species in the flora of Turkey, of which 10 of them are endemic [10]. The members of this genus have been widely used in traditional medicine and are greatly valued as spices and medicinal plants in Asia, especially in Iran, Turkey, and Iraq. The above-ground parts, the roots as well as the EOs of different species of this genus have both internal and external applications. The most popular indications of these plants are the alleviation of gastrointestinal symptoms, but various other uses have also been reported [9]. For instance, they are used as carminative, tonic, and anthelmintic agents, to heal scars and in the treatment of external bleeding, gastric or digestive disorders, wounds, and leuckoplakia. Moreover, *Prangos* species are known to act as stimulants, aphrodisiacs and natural fertilizers [9,11]. Based on the ethnobotanical uses of the members of the *Prangos* genus, several phytochemical studies have been performed and the presence of different groups of bioactive compounds including coumarins [12,13,14], essential oils [15,16], flavonoids [17,18] and phenolic acids [19] have been reported.

*P. uechtritzii* Boiss & Hausskn is a rigid and perennial plant. The leaves have long lobes and the flowers are yellow. The plant prefers rock limestone slopes and roadsides. It is generally distributed in the southeastern region of Turkey [20]. It is known as “deli çakşır” in local area of Turkey and has reputed aphrodisiac properties. In addition, the aerial parts of *P. uechtritzii* have been used to treat hemorrohoids in Anatolian folk medicine [14]. This plant’s antioxidant and antimicrobial effects as well as its chemical composition have been examined in earlier studies [14,21,22]. *P. meliocarpoides* Boiss. var. *meliocarpoides* is a perennial plant that ranges in height from 15 to 30 cm. It has yellow and glabrous flowers. It is distributed in the Inner Anatolia region of Turkey up to an altitude of 2000 m [20]. In earlier studies, the antioxidant properties of fruit extracts of the plant have been reported by several authors [21,23]. *P. heyniae* H. Duman & M.F. Watson is a perennial plant that reaches a height of 80 cm. Its flowers are yellow and glabrous. It is an endemic plant to the flora of Turkey and is distributed in the Central Anatolia region of Turkey, especially the calcareous slopes of Konya [24]. In recent studies, several coumarins and volatile compounds have been isolated from this plant, depending on the plant parts used [12,15].

In recent years, the number of studies reporting experimental data on the biological effects of *Prangos* species have increased considerably [17,22,25,26,27] and significant information has been gathered on the therapeutic properties of different species. Nevertheless, there are still a few species that have remained largely ignored with regard to certain aspects of their biological potentials, thereby reducing the possibility of their exploitation as phytomedicines. Therefore, in this study, the chemical composition, antioxidant and enzyme inhibitory effect of EOs extracted from three endemic *Prangos* species from Turkey (*P. heyniae*, *P. meliocarpoides* var. *meliocarpoides*, and *P. uechtritzi*.) were investigated and the molecular docking technique was applied to elucidate the binding interactions of selected EOs’ components with select enzymes.

## 2. Results and Discussion

### 2.1. Essential Oil Composition

The chemical composition of the EOs were analyzed using the gas-chromatography/mass spectrometry (GC/MS) and gas-chromatography-flame ionization detector (GC/FID). A total of 41 components was detected in *P. heyniae* EO (0.1–12.2%), while 40 components were found in *P. meliocarpoides* var. *meliocarpoides* EO (0.1–16.7%). On the other hand, only 30 components were identified in *P. uechtritzii* EO (0.1–24.6%) (Table 1). Ten compounds (*α*-pinene, *β*-pinene, sabinene, myrcene, limonene, *p*-cymene, *α*-copaene, *γ*-muurolene, caryophyllene oxide and spathulenol) were found to be common to all three EOs, although they varied in their percentages. As summarized in Table 2, *β*-bisabolenal (12.2%), caryophyllene oxide (7.9%), germacrene D (7.8%), elemol (7.4%) and *α*-humulene (6.7%) were the principal components of *P. heyniae* EO, while *P. meliocarpoides* var. *meliocarpoides* EO contained sabinene (16.7%), *p*-cymene (13.2%), bornyl acetate (11.8%), *α*-pinene (6.2%), *p*-cymen-8-ol (6.1%) as the major components. Moreover, *p*-cymene (24.6%), caryophyllene oxide (19.6%), 7-epi-1,2-dehydrosesquicineole (12.6%), limonene (3.2%) and *α*-bisabolol (3.2%) were present as the chief components of *P. uechtritzii* EO, accounting for 63.2% of the identified compounds (Table 2).

Previous studies have also analyzed the EOs of *Prangos* species under investigation herein. For instance, the bisabolene ether 7-epi-1,2-dehydrosesquicineole was also found to be present in the hydrodistilled fruit EO of *P. uechtritzii* as the major component (13.44%) in one study [28]. In another study, the EO of air-dried fruits of *P. uechtritzii* from East Anatolian region of Turkey was revealed to contain *α*-pinene (40.82%), nonene (17.03%), *β*-phellandrene (11.14%), *δ*-3-carene (7.39%), and *p*-cymene (4.90%) as principal components [22]. Furthermore, hydrodistilled EOs obtained from *P. heyniae* collected from four locations in Turkey were also studied for their chemical composition. The EOs were found to be rich in sesquiterpenes, germacrene D (10.3–12.1%), *β*-bisabolene (14.4%), kessane (26.9%), germacrene B (8.2%), elemol (3.4–46.9%), *β*-bisabolenal (1.4–70.7%), *β*-bisabolenol (8.4%) and an eudesmane type sesquiterpene (16.1%) which was later revealed to be 3,7(11)-eudesmadien-2-one [29]. Although, similar EO components were found to be present as previously reported in the EOs of the same species, they were found to vary. Indeed, EOs can vary greatly in their chemical composition both in qualitative and quantitative terms as they can be influenced by several factors including seasonal variations, plant organ, degree of maturity of the plant, geographic origin, and extraction method, among others [30]. The EOs derived from other *Prangos* species such as *P. pabularia* Lindl., *P. peucedanifolia* Fenzl., *P. ferulacea*, *P. platychlaena* and *P. pabularia* Lindl. have also been subject of investigations by other researchers [17,22,31,32]. 

### 2.2. Antioxidant Activity

Oxidative stress has been identified as the root cause of the development and evolution of several diseases. Supplementation of exogenous antioxidants or increasing endogenous antioxidant defenses of the body is a promising way of fighting the undesirable effects of reactive oxygen species (ROS) induced oxidative damage [33]. The plant kingdom is a rich source of health-promoting compounds especially as natural antioxidants [34]. Several studies have accentuated on the high antioxidant capacity of plants, their derivatives such as EOs and isolated compounds in the recent years, thus highlighting their usefulness in pharmaceutical, cosmetics, food and beverage industries, especially as some synthetic antioxidants such as BHA and BHT are now suspected to be potentially harmful to human health [35,36,37,38,39]. 

In the present investigation, the EOs extracted from all the three *Prangos* species were found to possess free radical scavenging potential in both DPPH and ABTS assays (0.43–1.74 mg TE/g and 24.18–92.99 mg TE/g). While *P. uechtritzii* EO showed the highest scavenging activity in DPPH assay, *P. heyniae* EO demonstrated the most significant scavenging activity in ABTS assay. Reducing potential was also noted by all EOs in CUPRAC and FRAP assays (103.15–113.43 mg TE/g and 47.98–61.20 mg TE/g, respectively) (Table 3). In FRAP assay also, *P. heyniae* EO displayed the highest activity while in CUPRAC assay *P. meliocarpoides* var. *meliocarpoides* EO showed higher reducing activity compared the other EOs. Metal chelating activity was demonstrated as well in the range of 28.66–30.94 mg EDTAE/g. Total antioxidant capacity was revealed by the phosphomolybdenum assay and the order of the potency of the EOs were as follows: *P. meliocarpoides* var. *meliocarpoides* > *P. heyniae* > *P. uechtritzii* (15.64–24.37 mmol TE/g) (Table 3).

Interestingly, several previous studies have also reported other members of the *Prangos* genus to exhibit strong antioxidant abilities [40,41]. In another study, the antioxidant activities of the water and methanol extracts obtained from the root, herb, and fruits of *P. ferulacea*, including the three species studied herein (*P. heyniae*, *P. meliocarpoides* var. *meliocarpoides*, and *P. uechtritzii*) from Konya Province (Turkey) were also evaluated using DPPH and thiobarbituric acid assays [21]. 

### 2.3. Enzyme Inhibitory Effects

Enzyme inhibitors play a significant role in the drug discovery process. An understanding of diseases at the molecular level has revealed the root cause of many to be the dysfunction, overexpression, or hyperactivation of enzymes. This hyperactivation or overexpression of enzymes can be treated by using suitable enzyme inhibitors. These efforts have provided several enzyme inhibitors in the clinic, including some from a natural origin [42]. Hence, in this present study, an attempt was made to assess the inhibitory effects of the EOs against some enzymes of clinical interest, notably cholinesterases, tyrosinase, amylase and glucosidase.

Cholinesterases are a group of serine hydrolases that split the neurotransmitter acetylcholine (ACh) and terminate its action. While acetylcholinesterase (AChE) plays the key role in ending cholinergic neurotransmission Butyrylcholinesterase (BChE) is a nonspecific cholinesterase enzyme that hydrolyzes choline-based esters. BChE plays a critical role in maintaining normal cholinergic function like AChE through hydrolyzing ACh [43]. Thus, cholinesterase inhibitors are useful substances that help to interfere with the break-down of ACh and prolong its action [44].

In this study, while no inhibitory activity was demonstrated by *P. meliocarpoides* var. *meliocarpoides* and *P. uechtritzii* EOs against both cholinesterases (AChE and BChE), while *P. heyniae* EO displayed only anti-BChE activity (9.85 mg GALAE/g) (Table 4).

The inhibition of *α*-glucosidase and *α*-amylase, enzymes involved in the digestion of carbohydrates, can significantly diminish the post-prandial surge of blood glucose and consequently can be an important strategy in the management of blood glucose level in type 2 diabetic and borderline patients. Presently, there is renewed interest in plant-based medicines and functional foods modulating physiological effects in the prevention and cure of diabetes and obesity. The plant kingdom is a wide field to search for natural effective oral hypoglycaemic agents that have minor or no side effects [45]. In the present study, the EOs were found to inhibit amylase (0.09–0.61 mmol ACAE/g) only, although the activity was not prominent, whereas they showed no activity against glucosidase (Table 4).

Tyrosinase plays a vital role because it is the critical enzyme and restriction enzyme in the course of melanin composition. Pigment spots and melanoma are markedly increased by cumulative tyrosinase activity and quantity. Consequently, tyrosinase inhibitors have received broad consideration owing to their use as hypopigmented agents in recent years [46].

Herein, all the EOs were found to act as tyrosinase inhibitors with inhibitory activity ranging from 46.34 to 69.56 mg KAE/g (Table 4). The anti-tyrosinase potency of the EOs was obtained in the following order: *P. meliocarpoides* var. *meliocarpoides* > *P. heyniae* > *P. uechtritzii*.

Another similar study was conducted but with an EO obtained from a different *Prangos* species, *P. gaubae* whereby the EO showed AChE (2.97 mg GEs/g oil), BChE (3.30 mg GEs/g oil), *α*-amylase (1.35 mmol ACEs/g oil), *α*-glucosidase (38.84 mmol ACEs/g oil), tyrosinase (29.24 mg KAEs/g oil) and lipase (1.59 mmol OEs/g oil) inhibitory activities. Additionally, strong antioxidant effects were observed in antiradical (DPPH and ABTS), reducing power (CUPRAC and FRAP), total antioxidant, as well as metal chelating assays [41]. On the other hand, in a recent study [40], methanol extracts of *P. ferulacea* (131.94 mg kojic acid (KAE) equivalent/g extract) and *P. peucedanifolia* (4.97 mmol acarbose equivalent (ACAE)/g extract) were reported to be potent inhibitors of tyrosinase and *α*-glucosidase, respectively. 

#### Multivariate Analysis

**Figure 1:**
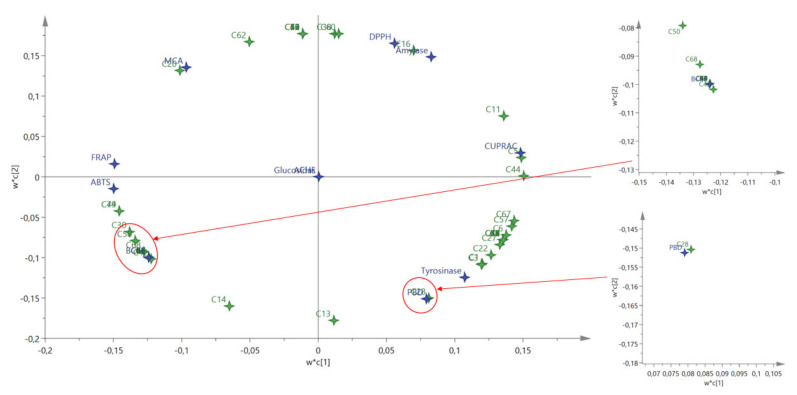
The biplot obtained from partial least squared (PLS) regression describing relationship between chemical compounds and bioactivities. For compounds numbers refer to Table 1.

**Figure 2:**
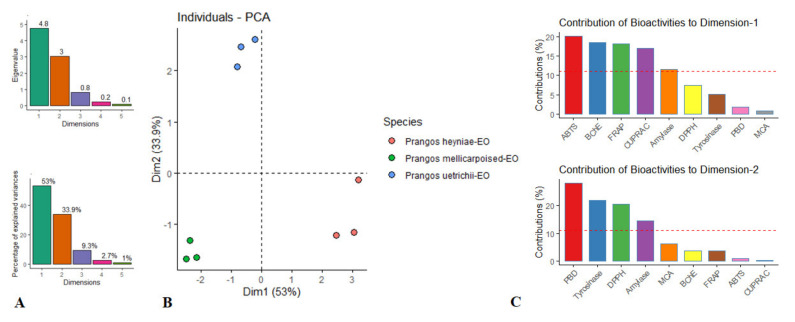
The principal component analysis on the biological activities of the EOs of Prangros species. (**A**). Eigenvalue and percentage of explained variances. (**B**). Score plot of dim1 and dim2 scores. (**C**). Corresponding bar plot representing influential bioactivities.

To gain more insights between the detected compounds and biological activities of the tested essential oils, we performed a PLS analysis. The results are given in Figure 1. R^2^X and Q^2^ values are indicator the quality of PLS parameters and the values were 0.87 and 0.97, respectively. Apparently, good connections were established between the identified compounds and biological activities. For example, *p*-cymene (compound **16**) made the main contribution to DPPH scavenging ability and this fact was confirmed by several researchers in previous studies [47,48]. As another example, *β*-elemene (compound **28**) was very close to the ability of phosphomolybdenum (PBD) and this compound has already been described as an agent for antioxidant therapy [49]. In metal chelating ability (MCA), *α*-copaene (compound **20**) was a major contributor. *β*-Pinene was very close in cupric reducing ability. Principal component analysis (PCA) was also performed to determine similarities or differences between the tested *Prangos* species based on their biological activities. The tested species were different and two components (PC1: 53% and PC2: 33.9%) were obtained in PCA (Figure 2). PC1 was mainly contributed by ABTS, BChE, FRAP and CUPRAC, while PBD, tyrosinase, DPPH and amylase were the main players in PC2. The obtained results could be useful for further application by using the tested species in future studies. 

### 2.4. Molecular Docking

**Figure 3:**
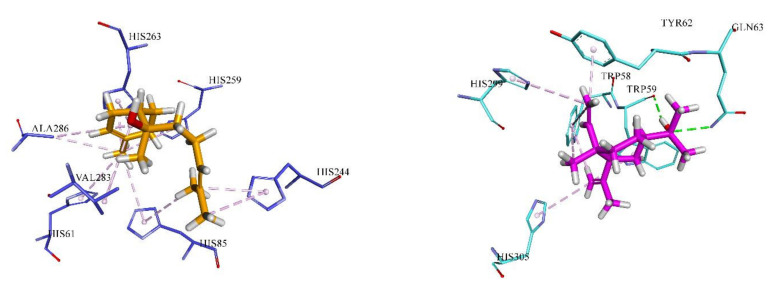
Three-dimensional binding interaction of tyrosinase (Blue stick) and α-bisabolol (golden stick). Amylase (Cyan stick model) and elemol (pink stick model). Hydrogen-bonding interactions are shown in green dash lines, while hydrophobic interactions are indicated in light pink dashed lines.

Molecular modelling has been used for the predicting the ligand–target affinity as well as interaction [50,51]. In this study, molecular docking investigation of 12 components (selected from five or the five most abundant components of each EO) from the three EOs of PH (*P. heyniae*), PM (*P. meliocarpoides* var. *meliocarpoides*) and PU (*P. uechtritzii*) against tyrosinase and amylase were investigated. The reason for selecting these two targeted enzymes was that these three EOs have demonstrated better inhibitory activity against these two enzymes. These EO components were selected to decipher the inhibition pattern against tyrosinase and amylase enzyme. Detailed docking scores of all selected compounds against tyrosinase and amylase are shown in Table 5 and details of the binding interactions of best docked pose of *α*-bisabolol and elemol against tyrosinase and amylase are reported in Table 6. Analysis of docking score revealed elemol and *α*-bisabolol to be the most effective in binding with tyrosinase and amylase based on their ChemGauss scores of −8.10 and −8.94, respectively. Detailed 3-D binding interactions of *α*-bisabolol against tyrosinase and elemol against amylase are depicted in Figure 3.

## 3. Materials and Methods

### 3.1. Plant Materials

*Prangos* species were collected in the city of Konya. The location information is given in Table 7 below. The plants’ identity were confirmed by one of co-authors (Evren Yıldıztugay) and voucher specimens have been deposited in Selcuk University. The aerial parts of the plant samples were dried under shade conditions for 10 days at room temperature. The plant samples were then powdered using a laboratory mill and the powdered plant samples were stored in the dark at room temperature.

### 3.2. Essential Oil Extraction and GC-MS Analysis

The essential oil was obtained by using the hydro-distillation technique. One hundred g dried plant samples were distilled in a Clevenger-type apparatus for 5 h. The essential oil was dried over sodium sulphate (anhydrous) and then the obtained essential oils were stored in an amber vial at +4 °C until analysis.

The obtained essential oil was characterized by gas chromatography-flame ionization detector (GC-FID) and gas chromatography-mass spectrophotometry (GC-MS) techniques. GC-MS analysis was performed by using a 5975 GC-MS system (Agilent, city, state abbreviation if USA, country) coupled to an Agilent 7890 A GC. To separate chemical components, a HP-Innowax column (60 m × 0.25 mm, 0.25 μm film thickness) was used. Other analytical parameters were reported in our earlier paper [52]. All analytical details are given in the Supplementary Materials.

The retention index (RI) calculated by co-injection with reference to a homologous series of *n*-alkanes (C_8_-C_30_) under identical experimental circumstances was used to identify the components. By comparing their mass spectra to those from the NIST 05 and Wiley 8th edition libraries, as well as comparing their RIs to literature values, we were able to make more accurate identifications.

### 3.3. Determination of Antioxidant and Enzyme Inhibitory Effects

The antioxidant activity of the essential oils tested in this study was determined using a variety of assays [53]. The assays used were 1,1-diphenyl-2-picrylhydrazyl (DPPH) and 2,2′-azino-bis(3-ethylbenzothiazoline)-6-sulfonic acid (ABTS) radical scavenging capacity (CUPRAC), ferric ion reducing antioxidant power (FRAP), metal chelating ability (MCA), and phosphomolybdenum assay (PDA). The data for the DPPH, ABTS, CUPRAC, and FRAP assays were expressed in mg Trolox equivalents (TE)/g essential oils whereas the data for MCA and PDA were expressed in mg EDTA equivalents (EDTAE)/g essential oils and mmol TE/g essential oils, respectively. Previously, we provided the experimental components for the acetylcholinesterase, butyrylcholinesterase, tyrosinase, *α*-amylase, and *α*-glucosidase assays. In cholinesterase assays, galanthamine was used as a positive control, and data were expressed as mg galanthamine equivalents (GALAE)/g essential oils. In the tyrosinase inhibitory assay, kojic acid was used as a standard inhibitor, and the results were expressed as mg kojic acid equivalents (KAE)/g essential oils [53,54]. In the anti-diabetic assays, acarbose was chosen as an inhibitor of both amylase and glucosidase, and the results are expressed as mmol acarbose equivalents (ACAE)/g essential oils. The experimental procedures of the assays are given in Supplementary Materials. The assays were performed in triplicate, and ANOVA (Tukey’s test) was used to determine the differences in the essential oils (*p* < 0.05).

### 3.4. Multivariate Analysis

A partial least squared (PLS) regression was performed for the relation between bioactive components and biological activities of the tested essential oils. A principal component analysis (PCA) was also performed to detect differences between the tested species. The multivariate analysis was done with the SIMCA 14.0 Software (Umetrics, Umeå, Sweden).

### 3.5. Molecular Docking

A molecular docking investigation of the 12 most abundant components from the three essential oils of *Prangos heyniae*, *Prangos meliocarpoides* var. *meliocarpoides* and *Prangos uechtritzii* against tyrosinase and amylase were carried out. A list of these compounds is summarized in Table 1.

The 2D structures of these selected compounds were retrieved from PubChem and then imported into Discovery studio client for making the 3D structures. OMEGA tool of OpenEye software was used to generate molecular structures of these compounds with their optimized stereoisomers, ring conformations, tautomers, and ionization states to make broad structural and chemical diversity from a single input compound. The default settings of OMEGA were used for the generation of multiconformers of each compound, as the generation of conformer is a prerequisite for subsequent molecular docking.

Three-dimensional X-rays crystallographic structures of the target enzymes tyrosinase and amylase were retrieved from the Enzyme Data Bank utilizing the PDB ID: 2Y9X and 4W93, respectively [55]. To prepare the enzyme structures, the Discovery Studio Client software was utilized, which eliminates the heteroatoms and water molecules, inserts hydrogens and missing residues and assigns charges. The binding site of each enzyme was located based on the co-crystalized ligand within each targeted enzyme. Prior to docking of compounds, optimization of docking calculations was performed by re-docking the co-crystal ligand within the active site of the respective enzyme. Binding site coordinates was obtained using a co-crystal ligand in the binding site of tyrosinase and amylase. In case of tyrosinase, active site was adjusted upon the coordinate X = −10.021, Y = −28.82, and Z = −43.596111 in XYZ dimensions. While in case of amylase, coordinate was selected using X = −9.63, Y = 4.34 and Z = −23.10 dimensions. In both cases of redocking co-crystal ligands within the target protein, the RMSD was found to be less than 2 Å which shows the reliability of the docking protocol [50].

After optimization of the docking protocol, molecular docking of all selected compounds (as listed in Table 1) were performed using the FRED tool in OEDOCKING of the OPENEYE Software. For each compound, ten poses were generated and sorted out based on the corresponding ChemGauss 4 score. The best-docked pose was selected based on the lowest ChemGauss 4 score for deciphering the binding interactions between the compound and the amino acid residues of the targeted enzyme. Discovery Studio Visualizer was used for the visualization of the binding interactions of the best compounds with the amino acid residues of tyrosinase and amylase.

## 4. Conclusions

In this study, the chemical profile of the EOs obtained from three endemic *Prangos* species from Turkey was revealed and analyzed. Moreover, the in vitro antioxidant and enzyme inhibitory properties of the EOs were investigated in an attempt to assess the biological potentials of the plant-derived products of these *Prangos* species. While all the EOs showed moderate to good antioxidant potentials, as evidenced by various assays, their activity as enzyme inhibitors was not as prominent. While all EOs demonstrated anti-tyrosinase and anti-amylase activity, only *P. heyniae* EO showed anti-BChE activity. Overall, the findings from this study unveiled the varying biological potentials of the studied EOs and some interesting prospects of the investigated EO components as enzyme inhibitors. Particularly, their antioxidant and anti-tyrosinase potentials offer suggestions for their use in the development of topical anti-hyperpigmentation formulations. However, further research is warranted to assess the potency and safety of these EOs and their active components as cosmeceutical agents in vivo.

## Figures and Tables

**Table 1:** Chemical composition of the tested *Prangos* essential oils.

No.	Compounds	RRI ^a^	PH (%)	PM (%)	PU (%)
1	*α*-Pinene	1023	1.6	6.2	0.4
2	*α*-Thujene	1026	-	0.3	-
3	Camphene	1068	0.3	1.4	-
4	Hexanal	1086	-	0.1	-
5	*β*-Pinene	1111	0.1	1.0	0.6
6	Sabinene	1124	0.1	16.7	0.8
7	*δ*-3-Carene	1157	-	0.4	-
8	Myrcene	1165	0.1	0.7	0.1
9	Heptanal	1189	0.1	-	-
10	Dehydro 1,8-cineole	1190	1.5	-	-
11	Limonene	1201	0.7	3.7	3.2
12	*β*-Phellandrene	1210	-	-	0.8
13	1,8-Cineole	1211	0.1	0.1	-
14	2-Pentylfuran	1234	0.2	0.1	
15	6-Methyl, 2-heptanone	1239	0.1	-	-
16	*p*-Cymene	1276	0.2	13.2	24.6
17	*α*, *p*-dimethylstyrene	1447	-	0.2	-
18	*α*-Cubebene	1465	0.2	-	-
19	*trans*-Sabinene hydrate	1469	-	1.0	-
20	*α*-Copaene	1501	1.4	0.4	2.0
21	*β*-Bourbonene	1531	1.1	-	-
22	Camphor	1535	0.2	1.5	-
23	*β*-Cubebene	1549	0.7	-	-
24	*cis*-Sabinene hydrate	1554	-	0.7	-
25	*trans*-Chrysanthenyl acetate	1581	-	0.4	-
26	Pinocarvone	1588	-	0.4	-
27	Bornyl acetate	1593	0.5	11.8	-
28	*β*-Elemene	1601	2.9	5.5	-
29	Terpinen-4-ol	1612	-	3.1	-
30	*β*-Caryophyllene	1614	3.8	-	0.8
31	*γ*-Elemene	1650	4.1	-	-
32	Myrtenal	1651	-	0.4	-
33	Sabina ketone	1655	-	0.9	-
34	*trans*-Pinocarveol	1670	-	0.9	-
35	*α*-Humulene	1689	6.7	-	-
36	*trans*-Verbenol	1690	-	4.2	-
37	Cryptone	1695	-	-	2.6
38	*γ*-Muurolene	1704	0.8	0.9	1.4
39	Germacrene D	1729	7.8	-	-
40	7-epi-1,2-Dehydrosesquicineole	1730	-	-	12.6
41	Verbonene	1732		1.4	-
42	*β*-Bisabolone	1738	5.7	0.1	-
43	Valencene	1740	-	-	0.5
44	*β*-Selinene	1743	-	0.9	0.4
45	Phellandral	1745	-	-	0.3
46	*α*-selinene	1747	-	0.2	-
47	Bicyclogermacrene	1754	0.3	-	-
48	Carvone	1757		1.0	-
49	*δ*-Cadinene	1773	2.0	-	0.7
50	*γ*-Cadinene	1779	0.7	-	0.1
51	Kessane	1785	-	2.5	-
52	*ar*-Curcumene	1787	-	-	0.5
53	*p*-Methylacetophenone	1800	-	-	0.3
54	Cumin aldehyde	1807	-	1.0	-
55	*trans*-Carveol	1846	-	0.9	-
56	Germacrene B	1856	3.3	-	-
57	*p*-Cymen-8-ol	1861	-	6.1	0.7
58	*α*-Calacorene	1943	-	-	0.3
59	1,5-Epoxysalvial-4(14)-ene	1947	1.8	-	-
60	4-Hydroxy-2-methylacetophenone	1950	-	2.8	15.1
61	Isocaryophyllene oxide	2002	-	-	1.7
62	Caryophyllene oxide	2017	7.9	3.5	19.6
63	Salvial-4(14)-en 1-one	2043	1.3	-	-
64	Humulene epoxide II	2074	4.0	-	-
65	Elemol	2095	7.4	-	-
66	*p*-Cresol	2101	-	-	0.5
67	Cumin alchol	2121	-	1.3	0.2
68	Spathulenol	2147	3.6	1.6	1.7
69	*γ*-Eudesmol	2187	2.7	-	-
70	*T*-Cadinol	2193	-	-	0.3
71	*T*-Muurolol	2208	2.3	-	-
72	*α*-Bisabolol	2232	-	-	3.2
73	*α*-Eudesmol	2246	1.0	-	-
74	*α*-Cadinol	2254	4.0	-	1.4
75	*β*-Eudesmol	2256	0.4	-	-
76	*β*-Bisabolenal	2377	12.2	-	-
	Total identified (%)		95.9	99.5	97.4

^a^ Relative retention indices calculated against n-alkanes. PH: *Prangos heyniae;* PM: *Prangos meliocarpoides* var. *meliocarpoides.* PU: *Prangos uechtritzii*.

**Table 2:** List if top most abundant selected compounds obtained from chemical profile from the three essential oils.

No.	Compounds	RRI ^a^	PH (%)	PM (%)	PU (%)
1	*α*-Pinene	1023	1.6	6.2	0.4
6	Sabinene	1124	0.1	16.7	0.8
11	Limonene	1201	0.7	3.7	3.2
16	*p*-Cymene	1276	0.2	13.2	24.6
27	Bornyl acetate	1593	0.5	11.8	-
35	*α*-Humulene	1689	6.7	-	-
39	Germacrene D	1729	7.8	-	-
40	7-epi-1,2-Dehydrosesquicineole	1730	-	-	12.6
57	*p*-Cymen-8-ol	1861	-	6.1	0.7
62	Caryophyllene oxide	2017	7.9	3.5	19.6
65	Elemol	2095	7.4	-	-
72	*α*-Bisabolol	2232	-	-	3.2
76	*β*-Bisabolenal	2377	12.2	-	-

^a^ Relative retention indices calculated against n-alkanes.

**Table 3:** Antioxidant Properties of the Tested Essential Oils.

Essentail Oils	DPPH (mg TE/g)	ABTS (mg TE/g)	CUPRAC (mg TE/g)	FRAP (mg TE/g)	MCA (mg EDTAE/g)	PBD (mmol TE/g)
*P. heyniae*	0.43 ± 0.01 ^c^	92.99 ± 1.29 ^a^	103.15 ± 3.69 ^b^	61.20 ± 0.73 ^a^	30.00 ± 5.82 ^a^	20.33 ± 0.48 ^b^
*P. meliocarpoides* var. *meliocarpoides*	1.01 ± 0.06 ^b^	24.18 ± 1.10 ^c^	113.43 ± 3.37 ^a^	47.98 ± 0.89 ^c^	28.66 ± 0.46 ^a^	24.37 ± 1.23 ^a^
*P. uechtritzii*	1.74 ± 0.10 ^a^	58.17 ± 1.46 ^b^	109.14 ± 1.00 ^a,b^	56.49 ± 0.64 ^b^	30.94 ± 0.20 ^a^	15.64 ± 0.28 ^c^

Values are reported as mean ± SD. TE: Trolox equivalent; EDTAE: EDTA equivalent; MCA: Metal chelating ability; PBD: Phosphomolybdenum assay. Different superscripts indicate significant differences in the tested essential oils (*p* < 0.05).

**Table 4:** Enzyme Inhibitory Effects of the Tested Essential Oils.

Essential Oil	AChE (mg GALAE/g)	BChE (mg GALAE/g)	Tyrosinase (mg KAE/g)	Amylase (mmol ACAE/g)	Glucosidase (mmol ACAE/g)
*P. heyniae*	na	9.85 ± 0.20	53.91 ± 2.11 ^b^	0.09 ± 0.01 ^c^	na
*P. meliocarpoides* var. *meliocarpoides*	na	na	69.56 ± 4.80 ^a^	0.41 ± 0.01 ^b^	na
*P. uechtritzii*	na	na	46.34 ± 6.51 ^b^	0.61 ± 0.01 ^a^	na

Values are reported as mean ± SD. GALAE: Galantamine equivalent; KAE: Kojic acid equivalent; ACAE: Acarbose equivalent; na: Not active. Different superscripts indicate significant differences in the tested essential oils (*p* < 0.05).

**Table 5:** Detailed binding score of the topmost compounds in three essential oils based on ChemGauss scores.

Compound Name	Binding Affinity Based on ChemGauss 4 Scores	
	Tyrosinase	Amylase
7-epi-1,2-Dehydrosesquicineole	−7.29	−7.38
Bornyl acetate	−6.95	−6.67
Caryophyllene oxide	−8.36	−7.45
Elemol	−7.33	**−8.10**
Germacrene D	−8.41	−7.23
Limonene	−8.68	−5.73
Sabinene	−7.64	−5.71
Pinene	−6.65	−5.39
*p*-Cymene	−7.79	−5.56
*p*-Cymen-8-ol	−7.18	−5.78
*α*-Bisabolol	**−8.94**	−8.03
*α*-Humulene	−7.78	−7.19
Reference (kojic acid)	−7.58	-
Reference (ascorbic acid)	-	−8.67

ChemGauss4 for topmost compound against each selected enzyme is shown in bold.

**Table 6:** Detailed binding Interaction of best docked pose of *α*-bisabolol and elemol against tyrosinase and amylase, respectively.

Interacting Amino Acid Residue of Tyrosinase and *α*-Bisabolol	Distance between Interacting Residue	Type of Bond
A:VAL283	4.7077	Alkyl
A:ALA286	5.3675	Alkyl
A:ALA286	4.4797	Alkyl
A:VAL283	4.2341	Alkyl
A:HIS61	4.7969	Pi-Alkyl
A:HIS85	4.9317	Pi-Alkyl
A:HIS85	5.0019	Pi-Alkyl
A:HIS244	4.3551	Pi-Alkyl
A:HIS244	4.6949	Pi-Alkyl
A:HIS259	5.2737	Pi-Alkyl
A:HIS263	3.8345	Pi-Alkyl
A:HIS263	3.7349	Pi-Alkyl
A:0TR410	4.3467	Pi-Alkyl
**Interacting Amino Acid residue of Amylase and Elemol**	**Distance between Interacting Residue**	**Type of Bond**
A:GLN63:NE2	2.9536	Conventional Hydrogen Bond
A:TRP59:O	2.123	Pi-Alkyl
A:TRP58	5.4757	Pi-Alkyl
A:TRP58	4.4585	Pi-Alkyl
A:TRP58	5.2415	Pi-Alkyl
A:TRP59	4.7261	Pi-Alkyl
A:TYR62	3.4596	Pi-Alkyl
A:HIS299	5.2942	Pi-Alkyl
A:HIS305	4.5785	Pi-Alkyl

**Table 7:** Locations and voucher numbers of the tested Prangos species.

*Prangos* Species	Locations	Voucher Numbers
*P. meliocarpoides* Boiss. var. *meliocarpoides*	Yavşan Location (Tuzgölü), Konya/Turkey, 905 m	EY-2998
*P. uechtritzii* Boiss & Hausskn	Between Hadim and Taşkent (2 km), Konya/Turkey, 1490 m	EY-3023
*P. heyniae* H. Duman & M.F. Watson	Between Hadim—Bozkır, Korualan location, Konya/Turkey, 1545 m	EY-3039

## Data Availability

Not applicable.

## Supplementary Materials

The following supporting information can be downloaded online.

## Sample Availability

Samples of the compounds are available from the authors.
