# Supplementary material for: Chemical Composition, Biological Activities and In Silico Analysis of Essential Oils of Three Endemic Prangos Species from Turkey
Source: Molecules. 2022 Mar 3;27(5):1676. doi: 10.3390/molecules27051676 (PMC8911840; doi:10.3390/molecules27051676)
Supplement: Supplementary file 1 [file molecules-27-01676-s001.zip › molecules-1613900-supplementary.pdf]

molecules

Supplementary Materials

Chemical Composition, Biological Activities and In Silico Analysis of Essential Oils of Three Endemic *Prangos* Species from Turkey

Gokhan Zengin [1], Mohamad Fawzi Mahomoodally [2], Evren Yıldıztugay [3], Sharmeen Jugreet [2], Shafi Ullah Khan [4], Stefano Dall'Acqua [5], Adriano Mollica [6], Abdelhakim Bouyahya [7] and Domenico Montesano [8,*]

1 Department of Biology, Science Faculty, Selcuk University, 42130 Konya, Turkey; gokhanzengin@selcuk.edu.tr
2 Department of Health Sciences, Faculty of Medicine and Health Sciences, University of Mauritius, Réduit 80837, Mauritius; f.mahomoodally@uom.ac.mu (M.F.M.); sharmeenjugs@gmail.com (S.J.)
3 Deparment of Biotechnology, Science Faculty, Selcuk University, 42130 Konya, Turkey; eytugay@gmail.com
4 Department of Pharmacy, Abasyn University, Peshawar 25000, Pakistan; shafiullahpharmd@gmail.com
5 Department of Pharmaceutical and Pharmacological Sciences, University of Padova, Via Marzolo 5, 35131 Padova, Italy; stefano.dallacqua@unipd.it
6 Department of Pharmacy, University "G. d'Annunzio" of Chieti-Pescara, 66100 Chieti, Italy; a.mollica@unich.it
7 Laboratory of Human Pathologies Biology, Department of Biology, Faculty of Sciences, Mohammed V University in Rabat, Rabat, 10106, Morocco; boyahyaa-90@hotmail.fr
8 Department of Pharmacy, University of Naples Federico II, Via D. Montesano 49, 80131 Naples, Italy
* Correspondence: domenico.montesano@unina.it

1. Chromatographic analysis

The oils were analyzed by gas chromatography-flame ionization detector (GC-FID) and gas chromatography/mass spectrophotometry (GC/MS) techniques. GC/MS analysis was carried out by an Agilent 5975 GC-MSD system coupled to an Agilent 7890A GC (Agilent Technologies Inc., Santa Clara, CA). HP-Innowax FSC column (60 m x 0.25 mm, 0.25 µm film thickness) was used with helium (purity 99.99 %) as carrier gas (1.2 mL/min). GC oven temperature was kept at 60 °C for 10 min and programmed to 220 °C at a rate of 4°C/min, and kept constant at 220 °C for further 10 min and then programmed to 240 °C at a rate of 1 °C/min. The split ratio was used at 40:1. The injector temperature was set to 250 °C and mass spectra were recorded at 70 eV. Mass range was adjusted from 35 to 450 m/z. GC-FID analysis was carried out by simultaneous auto-injection using Agilent 7693A series auto sampler; injections of 1 µL of the essential oils diluted with *n*-hexane (10 %, v/v) were used. Simultaneous autoinjection was provided equivalent retention times.

GC analysis was carried out using an Agilent 7890A GC system. In order to obtain the same elution order with GC/MS, simultaneous triplicate injections were done by using the same column and operational conditions. FID temperature was 300 °C.

Identification of constituents was achieved on the basis of retention index (RI) determined by co-injection with reference to a homologous series of *n*-alkanes (C_8-C_{30}), under same experimental conditions. Further identifications were carried out by comparison of their mass spectra with those from NIST 05 and Wiley 8th version and home-made MS library built up from pure substances and components of known EOs, as well as by comparison of their RIs with literature values.

2. Determination of Antioxidant and Enzyme Inhibitory Effects

Antioxidant (DPPH and ABTS radical scavenging, reducing power (CUPRAC and FRAP), phosphomolybdenum and metal chelating (ferrozine method) and enzyme inhibitory activities (cholinesterase (Elmann's method), tyrosinase (dopachrome method), α-amylase (iodine/potassium iodide method), α -glucosidase (chromogenic PNPG method) and pancreatic lipase (*p*-nitrophenyl butyrate (p-NPB) method) were determined.

For the DPPH (1,1-diphenyl-2-picrylhydrazyl):

Radical scavenging assay: Sample solution (0.5-5 mg/mL, 50 μL) was added to 150 μL of a 0.004% methanol solution of DPPH. The sample absorbance was read at 517 nm in a 96-well microplate after 30 min of incubation at room temperature in the dark. Trolox was used as a standard and a calibration curve was obtained by different trolox concentrations. DPPH radical scavenging activity was expressed as milligrams of trolox equivalents (mg TE/g extract).

For ABTS (2,2′-azino-bis(3-ethylbenzothiazoline) 6-sulfonic acid):

Radical scavenging assay: Briefly, ABTS+ was produced directly by reacting 7 mM ABTS solution with 2.45 mM potassium persulfate and allowing the mixture to stand for 12–16 min in the dark at room temperature. Prior to beginning the assay, ABTS solution was diluted in methanol to an absorbance of 0.700 ± 0.02 at 734 nm. Sample solution (0.5-5 mg/mL, 25 μL) was added to ABTS solution (200 μL) and mixed. The sample absorbance was read at 734 nm in a 96-well microplate after 30 min of incubation at room temperature. Trolox was used as a standard and a calibration curve was obtained by different trolox concentrations. The ABTS radical scavenging activity was expressed as milligrams of trolox equivalents (mg TE/g extract).

For CUPRAC (cupric ion reducing activity) activity assay:

Sample solution was added to premixed reaction mixture containing $CuCl_2$ (1 mL, 10 mM), neocuproine (1 mL, 7.5 mM) and NH_4Ac buffer (1 mL, 1 M, pH 7.0). Similarly, a blank was prepared by adding sample solution (25 μL, 0.5-5 mg/mL) to premixed reaction mixture (200 μL) without $CuCl_2$. Then, the sample and blank absorbances were read at 450 nm in a 96-well microplate after 30 min of incubation at room temperature. The absorbance of the blank was subtracted from that of the sample. Trolox was used as a standard and a calibration curve was obtained by different trolox concentrations. CUPRAC activity was expressed as milligrams of trolox equivalents (mg TE/g extract).

For FRAP (ferric reducing antioxidant power) activity assay:

Sample solution (25 μL, 0.5-5 mg/mL) was added to premixed FRAP reagent (200 μL) containing acetate buffer (0.3 M, pH 3.6), 2,4,6-tris(2-pyridyl)-S-triazine (TPTZ) (10 mM) in 40 mM HCl and ferric chloride (20 mM) in a ratio of 10:1:1 (v/v/v). Then, the sample absorbance was read at 593 nm in a 96-well microplate after 30 min of incubation at room temperature. Trolox was used as a standard and a calibration curve was obtained by different trolox concentrations. FRAP activity was expressed as milligrams of trolox equivalents (mg TE/g extract).

For phosphomolybdenum method:

Sample solution (100 μL, 0.5-5 mg/mL) was combined with 3 mL of reagent solution (0.6 M sulfuric acid, 28 mM sodium phosphate and 4 mM ammonium molybdate). The sample absorbance was read at 695 nm in a 96-well microplate after 90 min of incubation at 95 °C. Trolox was used as a standard and a calibration curve was obtained by different trolox concentrations. The total antioxidant capacity was expressed as millimoles of trolox equivalents (mmol TE/g extract).

For metal chelating activity assay:

Briefly, sample solution (100 μL, 0.5-5 mg/mL) was added to $FeCl_2$ solution (50 μL, 2 mM). The reaction was initiated by the addition of 5 mM ferrozine (100 μL). Similarly, a blank was prepared by adding sample solution (100 μL) to $FeCl_2$ solution (50 μL, 2 mM) and water (100 μL) without ferrozine. Then, the sample and blank absorbances were read at 562 nm in a 96-well microplate after 10 min of incubation at room temperature. The absorbance of the blank was subtracted from that of the sample. EDTA (disodium edetate) was used as a standard and a calibration curve was obtained by different EDTA concentrations The metal chelating activity was expressed as milligrams of EDTA equivalents (mg EDTAE/g extract).

For Cholinesterase (ChE) inhibitory activity assay:

Sample solution (100 μL, 0.5-5 mg/mL) was mixed with DTNB (5,5-dithio-bis(2-nitrobenzoic) acid, Sigma, St. Louis, MO, USA) (125 μL) and AChE (acetylcholinesterase (Electric ell acetylcholinesterase, Type-VI-S, EC 3.1.1.7,Sigma)), or BChE (butyrylcholinesterase (horse serum butyrylcholinesterase, EC 3.1.1.8, Sigma)) solution (25 μL) in Tris–HCl buffer (pH 8.0) in a 96-well microplate and incubated for 15 min at 25 °C. The reaction was then initiated with the addition of acetylthiocholine iodide (ATCI, Sigma) or butyrylthiocholine chloride (BTCl, Sigma) (25 μL). Similarly, a blank was prepared by adding sample solution to all reaction reagents without enzyme (AChE or BChE) solution. The sample and blank absorbances were read at 405 nm after 10 min of incubation at 25 °C. The absorbance of the blank was subtracted from that of the sample. Galanthamine was used as a standard and a calibration curve was obtained by different galanthamine concentrations. The cholinesterase inhibitory activity was expressed as galanthamine equivalents (mg GALAE/g extract).

For Tyrosinase inhibitory activity assay:

Sample solution (50 μL, 0.5-5 mg/mL) was mixed with tyrosinase solution (40 μL, Sigma) and phosphate buffer (100 μL, pH 6.8) in a 96-well microplate and incubated for 15 min at 25 °C. The reaction was then initiated with the addition of L-DOPA (40 μL, Sigma). Similarly, a blank was prepared by adding sample solution to all reaction reagents without enzyme (tyrosinase) solution. The sample and blank absorbances were read at 492 nm after 10 min of incubation at 25 °C. The absorbance of the blank was subtracted from that of the sample. Kojic acid was used as a standard and a calibration curve was obtained by different kojic acid concentrations. The tyrosinase inhibitory activity was expressed as kojic acid equivalents (mg KAE/g extract).

For α-amylase inhibitory activity assay:

Sample solution (50 μL, 0.5-5 mg/mL) was mixed with α-amylase solution (ex-porcine pancreas, EC 3.2.1.1, Sigma) (50 μL) in phosphate buffer (pH 6.9 with 6 mM sodium chloride) in a 96-well microplate and incubated for 10 min at 37 °C. After pre-incubation, the reaction was initiated with the addition of starch solution (50 μL, 0.05%). Similarly, a blank was prepared by adding sample solution to all reaction reagents without enzyme (α-amylase) solution. The reaction mixture was incubated 10 min at 37 °C. The reaction was then stopped with the addition of HCl (25 μL, 1 M). This was followed by addition of the iodine-potassium iodide solution (100 μL). The sample and blank absorbances were read at 630 nm. The absorbance of the blank was subtracted from that of the sample. Acarbose was used as a standard and a calibration curve was obtained by different acarbose concentrations. The α-amylase inhibitory activity was expressed as acarbose equivalents (mmol ACE/g extract).

For α-glucosidase inhibitory activity assay:

Sample solution (50 μL, 0.5-5 mg/mL) was mixed with glutathione (50 μL), α-glucosidase solution (from *Saccharomyces cerevisiae*, EC 3.2.1.20, Sigma) (50 μL) in phosphate buffer (pH 6.8) and PNPG (4-N-trophenyl-α-D-glucopyranoside, Sigma) (50 μL) in a 96-well microplate and incubated for 15 min at 37 °C. Similarly, a blank was prepared by adding sample solution to all reaction reagents without enzyme (α-glucosidase) solution. The reaction was then stopped with the addition of sodium carbonate (50 μL, 0.2 M). The sample and blank absorbances were read at 400 nm. The absorbance of the blank was subtracted from that of the sample. Acarbose was used as a standard and a calibration curve was obtained by different acarbose concentrations. The α-glucosidase inhibitory activity was expressed as acarbose equivalents (mmol ACE/g extract).
